# Kynurenine Pathway Metabolites in the Blood and Cerebrospinal Fluid Are Associated with Human Aging

**DOI:** 10.1155/2022/5019752

**Published:** 2022-10-21

**Authors:** Stein-Erik H. Solvang, Allison Hodge, Leiv Otto Watne, Otavio Cabral-Marques, Jan Erik Nordrehaug, Graham G. Giles, Pierre-Antoine Dugué, Ottar Nygård, Per Magne Ueland, Adrian McCann, Ane-Victoria Idland, Øivind Midttun, Arve Ulvik, Nathalie B. Halaas, Grethe S. Tell, Lasse M. Giil

**Affiliations:** ^1^Department of Internal Medicine, Haraldsplass Deaconess Hospital, Bergen, Norway; ^2^Department of Clinical Science, University of Bergen, Norway; ^3^Cancer Epidemiology Division, Cancer Council Victoria, Melbourne, VIC, Australia; ^4^Centre for Epidemiology and Biostatistics, Melbourne School of Population and Global Health, University of Melbourne, Parkville, VIC, Australia; ^5^Oslo Delirium Research Group, Department of Geriatric Medicine, Oslo University Hospital, Oslo, Norway; ^6^Department of Immunology, Institute of Biomedical Sciences, University of São Paulo, São Paulo, SP, Brazil; ^7^Department of Clinical and Toxicological Analyses, School of Pharmaceutical Sciences, University of São Paulo, São Paulo, Brazil; ^8^Network of Immunity in Infection, Malignancy, And Autoimmunity (NIIMA), Universal Scientific Education and Research Network (USERN), Sao Paulo, Brazil; ^9^Department of Pharmacy and Postgraduate Program of Health and Science, Federal University of Rio Grande do Norte, Natal, Brazil; ^10^Precision Medicine, School of Clinical Sciences at Monash Health, Monash University, Clayton, VIC, Australia; ^11^Department of Heart Disease, Haukeland University Hospital, Bergen, Norway; ^12^Bevital A/S, Bergen, Norway; ^13^Institute of Clinical Medicine, University of Oslo, Oslo, Norway; ^14^Research group for Lifespan Changes in Brain and Cognition, Department of Psychology, University of Oslo, Oslo, Norway; ^15^Department of Global Public Health and Primary Care, University of Bergen, Norway; ^16^Division of Mental and Physical Health, Norwegian Institute of Public Health, Bergen, Norway

## Abstract

The kynurenine pathway is implicated in aging, longevity, and immune regulation, but longitudinal studies and assessment of the cerebrospinal fluid (CSF) are lacking. We investigated tryptophan (Trp) and downstream kynurenine metabolites and their associations with age and change over time in four cohorts using comprehensive, targeted metabolomics. The study included 1574 participants in two cohorts with repeated metabolite measurements (mean age at baseline 58 years ± 8 SD and 62 ± 10 SD), 3161 community-dwelling older adults (age range 71-74 years), and 109 CSF donors (mean age 73 years ± 7 SD). In the first two cohorts, age was associated with kynurenine (Kyn), quinolinic acid (QA), and the kynurenine to tryptophan ratio (KTR), and inversely with Trp. Consistent with these findings, Kyn, QA, and KTR increased over time, whereas Trp decreased. Similarly, QA and KTR were higher in community-dwelling older adults of age 74 compared to 71, whereas Trp was lower. Kyn and QA were more strongly correlated with age in the CSF compared to serum and increased in a subset of participants with repeated CSF sampling (*n* = 33) over four years. We assessed associations with frailty and mortality in two cohorts. QA and KTR were most strongly associated with mortality and frailty. Our study provides robust evidence of changes in tryptophan and kynurenine metabolism with human aging and supports links with adverse health outcomes. Our results suggest that aging activates the inflammation and stress-driven kynurenine pathway systemically and in the brain, but we cannot determine whether this activation is harmful or adaptive. We identified a relatively stronger age-related increase of the potentially neurotoxic end-product QA in brain.

## 1. Introduction

Aging leads to chronic, low-grade inflammation and increases susceptibility to infections, a process referred to as immunosenescence, linked to morbidity and mortality [1]. Reflecting inflammaging, pro-inflammatory biomarkers increase with age including C-reactive protein (CRP), interleukin-6 (IL-6), tumor necrosis factor-alpha (TNF-*α*), and interferon-*γ* (IFN-*γ*) [2].

**Figure 1:**
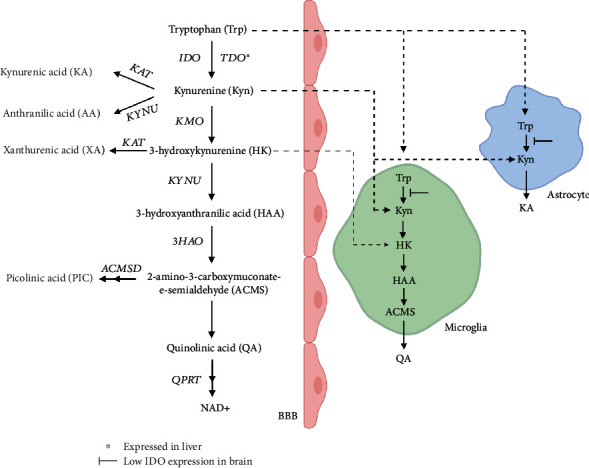
The kynurenine pathway and the blood-brain barrier. Indoleamine 2, 3 (IDO), and tryptophan 2, 3 dioxygenases (TDO) convert tryptophan to kynurenine (Kyn). Kynurenine monooxygenase (KMO) metabolizes Kyn to 3-hydroxykynurenine (HK), which is converted to 3-hydroxyanthranilic acid (HAA) by kynureninase (KYNU). HAA is the substrate of the enzyme 3-hydroxyanthranilic acid 3,4-dioxygenase (3HAO), which forms ACMS. ACMS spontaneously form QA, which is further metabolized by the action of quinolinate phosphoribosyl transferase (QPRT) and several intermediary steps to nicotinamide adenine dinucleotide (NAD^+^). Anthranilic acid (AA) is produced from Kyn by KYNU. Kynurenine aminotransferases (KATs) generate KA from Kyn and xanthurenic acid (XA) from HK. *α*-amino-*α*-carboxymuconate-*ε*-semialdehyde dehydrogenase (ACMSD) converts ACMS to aminomuconic semialdehyde, which is spontaneously converted to PIC. Trp, Kyn, and HK cross the blood-brain barrier (BBB), where KA is mainly synthesized in astrocytes and QA in microglial cells. Kyn is considered the primary precursor of kynurenines in the brain. The figure is based on [6].

Cytokines are mainly known for their ability to regulate the activity of the immune system, but they also regulate key metabolic pathways. Indeed, the interplay between metabolism and immunity also goes in the other direction as some metabolites affect immune cells [3]. The kynurenine pathway (KP), which degrades the essential amino acid tryptophan (Trp), is a metabolic pathway which is very closely intertwined with the immune system. The rate-limiting enzymes tryptophan 2,3 (TDO), and indoleamine 2,3 dioxygenases (IDO) generate kynurenine (Kyn) from Trp and are regulated by glucocorticosteroids and IFN-*γ*, respectively. Kyn gives rise to several metabolites referred to as kynurenines and finally to quinolinic acid (QA), the end-product of the KP [4] (Figure 1). Kyn can inhibit T effector cells, and natural killer, while stimulating T regulatory cells [5]. This pivotal KP metabolite also traverses the blood-brain barrier (BBB) where it can give rise to neuroactive metabolites such as QA and kynurenic acid (KA). Further, QA is a precursor of nicotinamide adenine dinucleotide (NAD+), which decreases with aging. Thus, the activity of the KP may have important consequences for both aging and immunosenescence.

Biomarkers have been used extensively to gain insights into human aging [6]. As proposed by Ingram et al., a biomarker of aging should show (1) cross-sectional correlation with age, (2) longitudinal change consistent with the cross-sectional correlation, and (3) be sensitive to small age differences [7]. A biomarker of aging should in addition be able to reflect clinical manifestations of aging, such as frailty, predict adverse health outcomes, or lifespan [8].

Previous studies have investigated the correlation between kynurenine metabolites and chronological age, but there have been some key limitations. Most studies have looked at only a few metabolites; thus, the knowledge of KP activity with age is restricted to select metabolites [9, 10]. Further, there have been no longitudinal studies, nor studies on small changes with age. Finally, the CSF studies have not been paired with blood samples [7, 11–19].

We evaluated associations between age and kynurenine concentrations in plasma, and changes in those concentrations over time, in two independent cohorts with repeated measurements. For comparison, we included the well-established biomarker of inflammaging, CRP [2]. In a third cohort, we evaluated variations in kynurenines when aged varied from 71 to 74 years. In a fourth cohort, we compared the strength of age-kynurenine correlations in sera to those in CSF and characterized changes in CSF kynurenines over time. Finally, we evaluated associations of kynurenines with frailty and mortality.

## 2. Material and Methods

### 2.1. Cohorts

#### 2.1.1. Melbourne Collaborative Cohort Study

The Melbourne Collaborative Cohort Study (MCCS) is an Australian prospective cohort study of 41,513 participants aged 27 to 76 years when they entered the study (see the supplementary material for details on enrollment). Participants were recruited between 1990 and 1994. At baseline, the participants answered questionnaires regarding demographics, lifestyle (alcohol consumption, diet, physical activity, and smoking), and medical history. At baseline, 67% of the participants provided fasting blood samples, while 33% provided non-fasting blood samples. After a median follow-up of 11 years, 90% of the participants provided fasting blood samples and 10% non-fasting. Blood was collected from all participants into sodium-heparin evacuated tubes (at baseline 1990-1994), centrifuged immediately (3000 rpm, 15 min, 20 °C), portioned into aliquots, and stored in liquid nitrogen at − 80 °C. Plasma samples were stored at − 80 °C until the time of analysis in 2018.

The first follow-up with new blood samples was performed between 2003 and 2007, where most measures taken at baseline were repeated for 26,824 participants. In the present study, a subset of 970 participants with repeated measurements (baseline and follow-up) of kynurenines in plasma were included. The selection of participants for metabolite measurements in MCCS was based on the presence of blood samples at both baseline and follow-up. Hence, none of these participants died between baseline and follow-up. For the survival analysis in MCCS, person-time at risk was counted from the follow-up visit, using the metabolite concentrations measured at this time point (see the supplementary material for ascertainment of vital status). All study participants provided informed consent per the Declaration of Helsinki [20]. The project was approved by the Human Research Ethics Committee of the Cancer Council Victoria.

#### 2.1.2. Western Norway B Vitamin Intervention Trial

The Western Norway B Vitamin Intervention Trial (WENBIT) is a prospective, double-blind, placebo-controlled secondary prevention study investigating the clinical effects of B vitamin intervention in patients who underwent coronary angiography for suspected coronary artery disease. The 3090 study participants were recruited at Haukeland University Hospital, Norway (January 2000–April 2004), and Stavanger University Hospital, Norway (September 2000–April 2004) [21]. All participants underwent routine clinical interviews and examinations before coronary angiography at baseline. Exclusion criteria were participation in other trials, unavailability for follow-up, known alcohol abuse, cancer, or severe mental illness. The participants were randomized to one of four arms: (1) vitamin B6, (2) B12/folate, (3) B12/folate/B6, and (4) placebo supplementations.

Study participants were scheduled for two follow-up visits. Blood samples were available from baseline and after the follow-ups conducted at a median of one and three years after baseline. There was also a clinical follow-up, described elsewhere [21]. The current study includes 604 patients with stable angina pectoris randomized to the placebo group with available, repeated non-fasting plasma samples. Samples were stored at − 80 °C until analysis in August 2007 to February 2008. The study protocol was approved by The Regional Committee for Medical and Health Research Ethics of Western Norway. All participants provided written informed consent.

#### 2.1.3. The Hordaland Health Study

The Hordaland Health Study (HUSK) is a community-based study of adults born 1925-1927, from the city of Bergen and three surrounding municipalities. The baseline examination was conducted from 1997 to 1999 (see the supplementary material for details on enrollment and registration of baseline data). For the present study, non-fasting plasma samples were available from 3161 participants, with follow-up data on mortality registered for up until 17 years. Blood samples were collected into EDTA-containing tubes. Samples were centrifuged after a maximum of 3 hours, and EDTA plasma was stored at − 80 °C until analysis in 2010. The study protocol was approved by The Regional Committee for Medical and Health Research Ethics of Western Norway. All participants provided informed, written consent.

#### 2.1.4. Elective Surgery Cohort (COGNORM Study)

The COGNORM study consists of 109 patients who underwent elective gynecological, orthopedic, or urological surgery and who following screening did not show signs of cognitive impairment. CSF and serum samples were collected per operatively during indication of spinal anesthesia. After 4 years, 33 participants volunteered for a second lumbar puncture. Cognitive screening protocols and further details on recruitment and sampling are given in the supplementary material. The study was conducted per the Declaration of Helsinki and approved by The Regional Committee for Medical and Health Research Ethics in Norway (REK 2011/2052). All participants provided informed, written consent.

### 2.2. Measurement of Metabolites and Biomarkers

The collection and storage of blood samples in MCCS, WENBIT, and HUSK have been described in detail previously [20–23]. In the COGNORM study, baseline fasting serum and CSF samples were collected at the onset of spinal anesthesia after an overnight fast before surgery, while participants were not fasting for the repeated CSF and serum samples [24]. Trp, Kyn, HAA, HK, KA, AA, XA, and QA were measured in all four cohorts. PIC was not measured in WENBIT. In the COGNORM study, CSF HAA and XA were below the limit of detection, and QA was not measured in WENBIT at the three-year follow-up. Trp and kynurenines were measured using liquid chromatography-tandem mass spectrometry (LC-MS/MS) [25], while CRP was determined using an immune matrix-assisted laser desorption/ionization mass spectrometry method [26]. LC-MS/MS combines the separating powers of liquid chromatography with the highly sensitive and selective mass spectrometry. Using authentic isotope-labelled internal standards allows for high precision analysis of low abundance biomarkers [27, 28]. We used an Agilent series 1100 HPLC system equipped with a thermostatted autosampler and degasser and an API 4000 triple-quadrupole tandem mass spectrometer from Applied Biosystems/MSD SCIEX with electrospray ionization (ESI) source for the biochemical analyses. Deproteinized plasma was injected into a Zorbax stable-bond C8 reversed-phase column. The mobile phase consisted of three components; (A) 650 mmol/L acetic acid, (B) 100 mmol/L heptafluorobutyric acid in component A, and (C) 90% acetonitrile in water. Analytes were eluted at a flow rate of 1.3 mL/min by the following gradient: 0–0.14 min (98% A and 2% B), 2.2 min (78% A, 2% B and 20% C), 2.3 min (60% A, 2% B and 38% C), 3.3 min (40% A, 2% B and 58% C), 3.4–4.1 min (2% B and 98% C), and 4.2–5.0 min (98%A and 2% B). Instrument parameters were as follows: ion spray (5500 V), curtain gas (10 psig), collision gas (4 psig), ion source temperature (650°C), and ion source gas 1 and 2 (75 psig) [25].

The ratio between Kyn and Trp (KTR) was defined as 100^∗^ Kyn (*μ*mol/L)/Trp (*μ*mol/L). The limit of detection for Trp was 0.4 *μ*mol/L, while the limit of detection for the kynurenines ranged from 0.5 nmol/L to 7.0 nmol/L. Within-day and between-day coefficients of variation for the kynurenines were 3.0–9.5% and 5.7-16.9%, respectively. For CRP, the limit of detection was 0.2 *μ*g/L, and within- and between-day coefficients of variation were 5.5–8.4% and 7.0–11.7%, respectively. All the biochemical analyses were performed by the laboratory of Bevital AS, Bergen, Norway (http://bevital.no).

### 2.3. Statistics

Metabolite concentrations are reported as the median and interquartile range due to skewness. We transformed all metabolite concentrations used in parametric analyses, except for Trp, using log-transformations, assessed normality using quantile-quantile plots, and subsequently scaled them using *z*-score so that effect sizes were comparable. Trp and the kynurenines served as outcomes in random intercept models (MCCS had two measurements) or random coefficient models (WENBIT had three measurements) in the two longitudinal cohorts with baseline age and time (in study) as predictors. In the MCCS, time was treated as a categorical variable (0 for baseline, 1 for follow-up), as there was a bimodal distribution of time (0 and around 11 years). The effect sizes were divided by 11 to obtain change per year. In WENBIT, we used a continuous variable representing years in the study. PIC was not measured in WENBIT and therefore omitted from the analysis as the purpose was to compare each metabolite in MCCS and WENBIT. Standardized fixed effects were calculated by multiplying the obtained fixed effects by the SD of the predictors (supplementary material). We used multinomial logit models to estimate the odds of being 72, 73, or 74 years old as compared to 71 years old, according to metabolite concentrations.

We used Cox regression for survival analyses, adjusted for age, sex, GFR, BMI, and smoking status adding CRP in a second model in MCCS and HUSK. Covariates were selected a priori based on their association with several kynurenine metabolites [15]. Associations with the frailty index (HUSK) were performed using standardized linear regression, adjusted for age and sex only (as GFR was part of the frailty index), adjusting for CRP in a final model. The frailty index by design ranges from 0 to 1, making log-transformation impossible. This was solved by multiplying the frailty index by 100 and by adding a constant of one, before log-transformation and standardization to a *z*-score.

In the CSF cohort, we used nonparametric Spearman Rho's to estimate the correlation between age and metabolites in serum, age, and metabolites in CSF and CSF-serum metabolite correlations, as sample size did not allow us to assume normality on the lognormal scale. We subsequently compared whether the correlations between age and the kynurenines were more substantial in the CSF or serum by testing the equivalence of paired correlation coefficients, as described by Cohen J. and Cohen P., implemented in the user-written Stata package “CORTESTI” [29]. The Wilcoxon sign-rank test was used to compare metabolite concentrations between baseline and follow-up in the subgroups with repeated CSF measurements. All statistical analyses were conducted using Stata (version 16, Stata Corp, College Station, Texas, US).

### 2.4. Frailty Index

Please see the supplementary material and Supplementary Table 1.

## 3. Results

### 3.1. Study Participants

#### 3.1.1. The Melbourne Collaborative Cohort Study (MCCS)

We included 970 study participants with a mean age at baseline of 57.6 (standard deviation (SD) 7.9) years (32% women) (Table 1). Concentrations of metabolites and CRP were measured in plasma samples taken at baseline and at follow-up after a median of eleven years, with mortality registered until 16 years after the last follow-up.

#### 3.1.2. The Western Norway B Vitamin Intervention Trial (WENBIT): Placebo Group

The patients with stable angina pectoris (SAP, *n* = 604) had a mean age of 61.9 (SD 9.7) years at baseline, where 22.9% were women (Table 1). Metabolites were measured in plasma at baseline and at follow-ups after a median of one and three years.

#### 3.1.3. The Hordaland Health Study (HUSK)

We included 3161 community-dwelling participants (44.3% women) aged 71-74 years with available blood-samples (Table 1) with mortality registered up until 17 years after baseline. Cognitive testing was performed in 2152 participants, of which 1691 also had available data to estimate a frailty index.

#### 3.1.4. Elective Surgery Cohort (COGNORM Study)

Patients (*n* = 109) undergoing elective surgery (mean age of 73.3 years (SD 6.8), 46% women) with available CSF and serum samples were included from the COGNORM study (Table 1). After four years, the participants were invited to donate a second CSF sample. Thirty-three participants volunteered for puncture and had available repeated CSF samples for this study.

All metabolite and CRP concentrations for the cohorts are reported in Supplementary Table 2.

### 3.2. Associations between Kynurenines and Age

**Figure 2:**
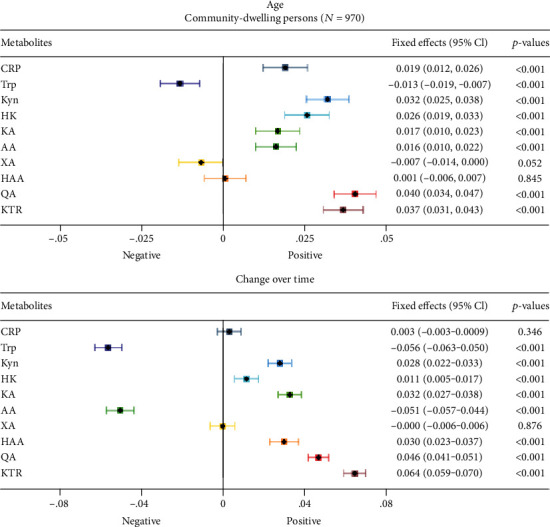
Change in plasma metabolites of the kynurenine pathway with age (upper panel) and over time (lower panel) in community-dwelling persons (MCCS study). The mean age of MCCS participants was 57.6 years at baseline, with follow-up after a median of 11 years. Log-transformed and standardized metabolites (and CRP for comparison) were entered as outcomes in linear mixed-effects models with baseline age and time as predictors. In the model, time was a categorical variable, and the effect sizes for the time were divided by 11 to yield change per year. The fixed effects reflect a one standard deviation change in the standardized, log-transformed metabolite per year of chronological age or per year in the study. Of note, the *x*-axes for the fixed effects are on different scales. Abbreviations: AA: anthranilic acid; CRP: C-reactive protein; HAA: 3-hydroxyanthranilic acid; HK: 3-hydroxykynurenine; KA: kynurenic acid; KTR: kynurenine to tryptophan ratio; Kyn: kynurenine; MCCS: Melbourne Collaborative Cohort Study; *p*: *p* value; Trp: tryptophan; QA: quinolinic acid; XA: xanthurenic acid; 95% CI: 95% confidence interval.

**Figure 3:**
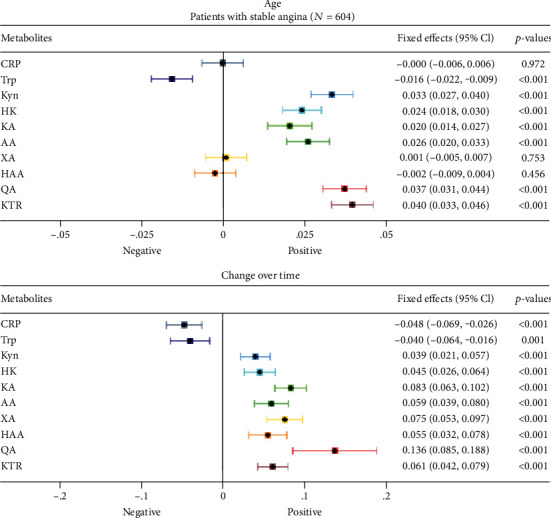
Change in plasma metabolites of the kynurenine pathway with age (upper panel) and over time (lower panel) in patients with stable angina pectoris (WENBIT study). The mean age of the patients was 61.9 years at baseline, and they were followed up after 1 and 3 years. Metabolites (and CRP) were log-transformed, standardized, and entered as outcomes in linear mixed-effects models with age at the study baseline and time (years in the study) as predictors. The fixed effects reflect a one standard deviation change in the standardized, log-transformed metabolite per year of chronological age or per year in the study. Of note, the *x*-axes for the fixed effects are on different scales. Abbreviations: AA: anthranilic acid; CRP: C-reactive protein; HAA: 3-hydroxyanthranilic acid; HK: 3-hydroxykynurenine; KA: kynurenic acid; KTR: kynurenine to tryptophan ratio; Kyn: kynurenine; *p*: *p* value; Trp: tryptophan; QA: quinolinic acid; WENBIT: Western Norway B Vitamin Intervention Trial; XA: xanthurenic acid; 95% CI: 95% confidence interval.

Figure 2 (MCCS) and Figure 3 (WENBIT) summarize the fixed effects (FE) from a multilevel model, which represents a one SD change in biomarkers per year of age or time in study.

Supplementary Table 3 lists standardized fixed effects. In MCCS, the strongest associations with age were found for QA (FE 0.040 [0.034, 0.047], *p* < .001), KTR (FE 0.037 [0.031, 0.043], *p* < .001), and Kyn (FE 0.032 [0.025, 0.038], *p* < .001). The estimated associations with age were weaker for 3-hydroxykynurenine (HK), CRP, KA, and anthranilic acid (AA). In contrast, Trp was inversely associated with age. In WENBIT, KTR (FE 0.040 [0.033, 0.046], *p* < .001), QA (FE 0.037 [0.031, 0.044], *p* < .001), and Kyn (FE 0.033 [0.027, 0.040], *p* < .001) displayed the strongest associations with age. There were weaker associations between age, AA, HK, and KA. Again, Trp was inversely associated with age.

### 3.3. Changes in Kynurenine Concentrations Over Time

Samples in MCCS were taken at baseline and again after a median of 11 years. We identified an increase in KTR (FE 0.064 [0.059, 0.070], *p* < .001) and QA (FE 0.046 [0.041, 0.051], *p* < .001) over time, with smaller increases in KA, HK and HAA. Trp (FE − 0.056 [− 0.063, − 0.050], *p* < .001) and AA (FE − 0.051 [− 0.057, − 0.044], *p* < .001) decreased over time.

Samples in WENBIT were taken at a median of one and three years after baseline. Here, QA (FE 0.136 [0.085, 0.188], *p* < .001) and KTR (FE 0.061 [0.042, 0.079], *p* < .001) increased. However, QA was measured only at baseline and after one year. HK, KA, AA, HAA, and XA increased to a lesser extent over time, whereas Trp decreased. Supplementary Figure 1 illustrates how KTR varies with age and over time. By comparison, CRP showed minor associations with age and inconsistent changes over time in MCCS and WENBIT.

### 3.4. Metabolite concentrations in persons aged 71 to 74 years in HUSK

Trp was lower in persons 74 years old compared to 71 (Odds ratio (OR) 0.86 [0.76, 0.98], *p* = .019). In contrast, HK (OR 1.26 [1.11, 1.42], *p* < .001), KTR (OR 1.17 [1.03, 1.33], *p* = .012), and QA (OR 1.16 [1.02, 1.31], *p* = .020) were significantly higher. CRP (OR 1.12 [0.98, 1.27], *p* = .084) did not reach statistical significance. QA was also higher in persons 72 or 73 years old compared to 71 years old (Supplementary Table 4).

### 3.5. Aging and Kynurenine Pathway Metabolites in the Cerebrospinal Fluid

In the 109 participants of the COGNORM study, the metabolites in serum correlated moderately (Trp, HK, and KA) to strongly (Kyn, picolinic acid (PIC), and QA) with the corresponding CSF metabolites. The correlation (Spearman Rho's (rs)) between metabolites and age was significantly stronger in the CSF compared to serum for QA (CSF: rs 0.55; serum: rs 0.37) and Kyn (CSF: rs 0.39; serum: rs 0.24). There was a similar trend of stronger correlation in CSF for KA (CSF: rs 0.32; serum: rs 0.15) but not for AA (CSF: rs 0.23; serum: rs 0.23). For Trp, the correlation with age was positive in CSF (rs 0.14) but negative in serum (rs − 0.27) (Table 2(a)). Compared to the first age quartile, CSF-QA concentrations doubled in the fourth quartile, and CSF QA as a percentage of serum QA also increased with age (Table 2(b)). HK and PIC were not correlated with age in either serum or CSF. In the subgroup of 33 participants with a second lumbar puncture after four years, there was an increase in the CSF of QA (median: 31.2 to 42.6 nmol/L, *p* < .001) and Kyn (51.4 to 57.7 nmol/L, *p* < .001), whereas HK increased marginally (4.4 to 4.9 nmol/L, *p* = 0.038). There was no significant change in Trp, KA, AA, and PIC (Table 2(c)).

### 3.6. Kynurenine Concentrations and Frailty in HUSK

Using the frailty index score as the outcome, CRP was the metabolite with the strongest association with frailty (*β* 0.19 [0.14, 0.22], *p* < .001) adjusted for age, sex, and smoking status. Significant associations with frailty were identified for several kynurenines: QA (*β* 0.18 [0.14, 0.22], *p* < .001), Kyn (*β* 0.15 [0.11, 0.19], *p* < .001), KTR (*β* 0.15 [0.11, 0.19], *p* < .001), HK (*β* 0.15 [0.10, 0.19], *p* < 0.001), KA (*β* 0.08 [0.3, 0.11], *p* < 0.001), and HAA (*β* 0.10 [0.06, 0.15], *p* < 0.01). These associations were only slightly attenuated after adjustment for CRP and remained significant (Table 3).

### 3.7. Kynurenines as Predictors of All-Cause Mortality

Adjusted for age, sex, BMI, smoking status, GFR, and CRP, higher QA (HUSK: HR 1.14 [1.07, 1.20], *p* = <.001, MCCS: HR 1.28 [1.12, 1.47], *p* < .001) and KTR (HUSK: HR 1.21 [1.14, 1.28], *p* < .001, MCCS: HR 1.13 [1.01, 1.28], *p* = .047) concentrations were associated with higher all-cause mortality in the two cohorts. Higher Trp concentrations were associated with lower all-cause mortality (HUSK: HR 0.91 [0.86, 0.96], *p* < .001, MCCS: HR 0.87 [0.79, 0.97], *p* = .010). Adjusted for age, sex, BMI, smoking status, GFR, and KTR, higher CRP concentrations were associated with higher all-cause mortality in both cohorts (HUSK: HR 1.06 [1.00, 1.11], *p* = .039, MCCS: HR 1.20 [1.08, 1.34], *p* < .001). Several of these results have been previously published (Supplementary Table 5) [30, 31].

## 4. Discussion

Our studies of multiple cohorts with longitudinal measurements show that in particular, Trp depletion with accumulation of Kyn and the KP end-product QA are integral parts of human aging, perhaps more substantially so in the brain. The immunoregulatory and neuroactive properties of these metabolites raises intriguing questions as to whether they participate in parts of the aging process, and their potential relevance was highlighted by association with both frailty and mortality.

There was a consistent inverse association between age and Trp and positive associations between age and Kyn, HK, KA, QA, and KTR in community-dwelling persons (MCCS) and patients with stable angina pectoris (WENBIT). Changes in metabolite concentrations over time and associations with age were concordant in both MCCS and WENBIT, where Trp decreased and Kyn, KA, HK, QA, and KTR increased. Similarly, community-dwelling HUSK participants 74 years of age had lower Trp than 71-year-olds, accompanied by higher HK, QA, and KTR. The findings for HAA and AA were inconsistent (HUSK, MCCS, and WENBIT). The increase in AA over time in WENBIT, in contrast to a decrease in MCCS, could be due to its association with ischemic heart disease [32]. Several previous cross-sectional studies have identified lower Trp [4, 13, 19] and higher Kyn in older persons [13, 19, 33, 34], and our study corroborates these findings. Using comprehensive profiling of the kynurenine pathway, we found that aging was most strongly related to an increase in KTR and QA.

In MCCS, the association of age with QA and KTR was approximately two-fold the strength of the association observed between age and CRP. This finding is somewhat surprising, as IL-6, which stimulates CRP synthesis, increases more with age than IFN-*γ*, which induces IDO [35]. We did not find consistent changes in CRP over time, nor an association with age (MCCS, WENBIT). There was a decline in CRP in WENBIT, which may be related to lipid-lowering treatment in coronary artery disease [36], thus concealing the natural course of CRP.

Aging macrophages produce more cytokines and display more IDO activity [5]. Trp typically decreases with inflammation, as IDO activation generates Kyn and downstream metabolites [11]. Human monocytes stimulated by IFN-*γ* ex vivo display increased levels of QA and KTR [37]. Thus, the pattern of changes in kynurenines observed in our data could be related to senescent macrophages. Alternatively, TDO activation with age could also affect kynurenines as glucocorticoids, which induce TDO, increase with age and NADPH, which decreases with age, inhibits TDO [38–40]. However, TDO is substrate activated by Trp [39] which decreased with aging. Inflammation-driven activation of the kynurenine pathway paradoxically inhibits de novo NAD^+^ generation from QA in macrophages and may impair resolution of inflammation [41]. The expression of the enzyme quinolate phosphoribosyl transferase (QPRT) (which metabolizes QA) decreases in aging rats [42]. Thus, reduced QA degradation is a potential explanation for the relative abundance of QA with aging observed in this study.

Altered concentrations of kynurenines could affect the aging immune system. It has been suggested that low Trp and high kynurenines could serve to constrain chronic inflammation [11] possibly by suppressing CD8^+^ and CD4^+^ T-cells, and stimulate Tregs [43] [11, 44–46]. However, our study cannot determine whether activation of the kynurenine pathway in human aging is harmful or adaptive. On the one hand, the increase in kynurenine metabolites following IDO activation due to chronic inflammation could lead to more oxidative stress, as several kynurenines lead to higher levels of oxidative stress [47]. Further, higher concentrations of the potentially neurotoxic QA could drive neurons closer to apoptosis. On the other hand, as kynurenines can suppress the immune response, inflammation might have been worse had it not been for the immunosuppresive actions of elevated kynurenines. Further, the contribution of QA to de novo NAD+ synthesis might be beneficial if NAD+ deficiency, more prevalent with aging, is present [47].

Our findings suggest that age-related kynurenine pathway activation may be more pronounced in the brain than systemically, as indicated by stronger correlations of age with Kyn, KA, and QA in the CSF compared to serum. Kyn and QA and marginally HK also increased in the CSF over time, expanding upon previous studies on CSF measurements at one time point [10, 48]. Trp correlated inversely with age in serum, with a nonsignificant positive correlation in the CSF, with previous studies finding no correlation or inverse correlation in women [48, 49]. KA correlated with age in line with a previous study [10], but did not change over time raising the possibility that KA could be correlated with age due to cohort effects, disease, or medication which are often stable factors over the span of four years. CSF-QA was the most strongly correlated to age.

Kynurenines in the CSF would be expected to increase with higher systemic levels and with microglial activation. Our study cannot determine why there is a relative abundance of kynurenines in the aging brain, but the combination of increased systemic production and aging microglia likely contributes [50]. Hallmarks of brain aging such as oxidative stress, impaired proteostasis, and mitochondrial dysfunction increases the neuronal vulnerability to excitotoxic damage [51]. Studies have indicated that QA can produce reactive oxygen species by forming a complex with iron, leading cells to undergo oxidative damage as part of their degradation [52]. Further, increasing basal concentrations of the NMDA receptor agonist QA, without a similar increase in the NMDAR antagonist KA, could be relevant for diseases where excitotoxicity is important such as neurodegenerative disease [53]. Indeed, patients with neurodegenerative and psychiatric disorders have increased concentrations of kynurenines in the CSF and brain tissue [54].

Frailty is a hallmark of pathological aging [6] and, here, measured using a frailty index [55]. In 71-74 years old community-dwelling persons in HUSK, QA was the kynurenine most strongly associated with frailty, closely followed by Kyn and KTR, adjusted for sex and age. CRP, however, was more strongly associated with frailty than these kynurenines. HK, KA, and HAA were also significantly related to frailty. These findings largely correspond to the metabolites that were the most altered with aging in HUSK, MCCS, and WENBIT. Previous studies have also identified associations between higher KTR and frailty [56, 57]. Our study highlights the importance of QA, although our method of measuring frailty differs from the previous studies [56, 57].

Compared to other kynurenines, QA and KTR displayed relatively stronger associations with mortality in MCCS and HUSK independently of age, sex, renal function, BMI, and CRP. Higher plasma Trp concentrations were associated with improved survival in both MCCS and HUSK. The associations of the kynurenines and CRP with mortality in HUSK and MCCS have been published previously [30, 31]. Higher KTR has been associated with coronary events, cancer, frailty, and mortality in nonagenarians [11]. Experimental studies suggest mechanistic links between Trp, kynurenines, and longevity. In *C. elegans* and *D. melanogaster*, depletion or loss of function of TDO results in increased longevity [58, 59]. Collectively, our observations suggest that KTR and QA are associated with pathological aging independent of CRP.

Future studies should investigate whether KTR and QA can predict phenotypic aging such as frailty. Perhaps more importantly, studies are needed to determine if and how Trp, Kyn, and other immunoactive kynurenines affect immunosenescence. Finally, the twofaced roles of QA as a potential excitotoxin at higher concentrations and as a precursor of NAD+ that may be important with lower NAD+ concentrations should be scrutinized. In the future, one might envision biomarkers that are highly associated with age such as KTR and QA to be used as risk markers or rather be part of comprehensive biomarker-based aging panels to identify individuals at risk of pathological aging. If evidence is found that the KP contributes to aging or inflammaging, enzyme inhibitors are already available.

Strengths of the study include using four independent cohorts from different populations with cross-sectional and longitudinal data, relatively large sample sizes, both serum/plasma and CSF measurements, and centralized, comprehensive, targeted profiling of kynurenines. The main limitations are nonstandardized and different procedures for the collection and storage of samples between cohorts, including differences in fasting and non-fasting samples which can impact Trp concentrations (see material and methods). There were differences in follow-up times between the cohorts with repeated metabolite measurements. This makes exact comparisons of the rates of change in metabolites concentrations difficult. We did not aim to investigate why the concentrations of kynurenines increase with age where declining renal function and inflammation likely contribute [60]. The kynurenines are mostly stable in frozen serum or EDTA plasma samples under long-term storage, although anthranilic acid can increase over time [61, 62]. However, long-term stability has not been investigated in the CSF. Further, although long-term stability has been investigated, we cannot rule out an influence from long-term storage, as the storage times in these studies were rather long, especially for the MCCS cohort. However, the consistency of findings across cohorts and between cross-sectional and longitudinal changes support the findings from these studies.

In conclusion, our findings of Trp depletion with elevated Kyn corroborates previous studies of the KP as an integral part of human aging, but we importantly find that the most typical change in the KP in human aging is an increase in QA which is linked to oxidative stress and neurotoxicity. Importantly, QA elevation with age seems to be more pronounced in the CSF. Our comprehensive study establishes substantial alterations of immune (Kyn) and neuroactive (QA) kynurenines in human aging.

## Figures and Tables

**Table 1:** Characteristics of the study participants in the four cohorts.

MCCS: community-dwelling persons (*N* = 970)	
Age at baseline, years, mean (SD)	57.6 (7.9)
Time until follow-up, years, median (IQR)	11.3 (1.7)
Women, %	32
GFR^a^, mean (SD)	
Baseline	89.2 (20.2)
Follow-up^b^	77.6 (20.1)
Survival	
Follow-up, years^c^	16.2
Events, *n*	308
Mortality rate, per 1000 person-years	25.0
WENBIT: patients with stable angina pectoris (*N* = 604)	
Age at baseline, years, mean (SD)	61.9 (9.7)
Time until first follow-up, years, median (IQR)	1.07 (0.13)
Time until second follow-up, years, median (IQR)	3.27 (1.29)
Women, %	22.9
GFR^d^, mean (SD)	75.2 (14.7)
HUSK: community-dwelling persons (*N* = 3161)	
Age, %	
71	17.9
72	34.4
73	33.3
74	14.3
Women, %	44.3
GFR^c^, mean (SD)	72.4 (15.8)
Survival	
Follow-up, years^c^	16.7
Deaths, *n*	1674
Mortality rate, per 1000 person-years	39.2
Subgroup with frailty score (*n* = 1691)	
Age, %	
71	15.8
72	33.8
73	32.8
74	17.6
Women, %	46.4
GFR^d^, mean (SD)	72.4 (12.7)
Frailty score, median [IQR]	0.26 [0.81]
COGNORM study: cognitively normal persons (*N* = 109)	
Age, years, mean (SD)	73.3 (6.8)
Women, %	46

Abbreviations: HUSK: Hordaland Health Study; MCCS: Melbourne Collaborative Cohort Study; GFR: glomerular filtration rate; IQR: interquartile range; SAP: stable angina pectoris; SD: standard deviation; WENBIT: Western Norway B Vitamin Intervention Trial. ^a^Calculated based on the cystatin C equation [52]. ^b^The follow-up after a median of 11 years constituted the baseline for mortality analyses in MCCS. ^c^The longest time from inclusion to censoring. ^d^Calculated based on the creatinine 4-variable modification of diet in renal disease equations [45].

**Table:** (a) Serum and cerebrospinal fluid correlations with age for tryptophan and kynurenines in 109 cognitively healthy persons undergoing elective surgery (COGNORM)

	Correlations between Metabolites^a^	Correlations between metabolites and age^a^		Difference in serum vs. CSF correlations with age^b^
	Serum vs. CSF	Serum	CSF	Null-hypothesis of equivalence
Trp	0.26^∗^	− 0.27^∗^	0.14	.001^∗^
Kyn	0.68^∗∗^	0.24^∗^	0.39^∗∗^	.039^∗^
HK	0.40^∗∗^	0.17	0.17	.999
KA	0.28^∗^	0.15	0.32^∗∗^	.136
AA	0.36^∗∗^	0.29^∗^	0.23^∗^	.574
PIC	0.70^∗∗^	− 0.11	0.03	.067
QA	0.78^∗∗^	0.37^∗∗^	0.55^∗∗^	.001^∗^

**Table:** (b) Quinolinic acid concentrations in nmol/L according to age quartiles

	Serum Median (IQR)	CSF Median (IQR)	% in CSF vs. serum Median (IQR)
64-68	373 (158)	29.4 (10.1)	7.95 (2.62)
69-71	398 (267)	33.0 (20.3)	8.74 (2.64)
72-77	429 (135)	42.7 (16.1)	8.75 (4.28)
78-91	647 (723)	65.9 (65.1)	10.6 (3.52)

**Table:** (c) Change in cerebrospinal fluid kynurenines over four years (*n* = 33)

	Baseline Median (IQR)	Four-year follow-up Median (IQR)	_p_e
Trp^c^	2.64 (0.7)	2.64 (0.6)	.774
Kyn^d^	51.4 (14.2)	57.7 (29.9)	<.001^∗∗^
HK^c^	4.42 (2.27)	4.91 (2.45)	.038^∗^
KA^c^	2.47 (1.33)	2.25 (1.13)	.816
AA^c^	9.48 (7.18)	10.9 (2.88)	.788
PIC^c^	19.2 (8.2)	18.9 (7.4)	.469
QA^d^	31.2 (20.3)	42.6 (24.7)	<.001^∗∗^

Note: CSF HAA and XA were under the limit of detection and thus omitted from this analysis. Abbreviations: AA: anthranilic acid; CSF: cerebrospinal fluid; HK: 3-hydroxykynurenine; IQR: interquartile range; KA: kynurenic acid; Kyn: kynurenine; *p*: *p* value; PIC: picolinic acid; Trp: tryptophan; QA: quinolinic acid. ^a^Spearman rho correlation coefficients. ^b^Equivalence of correlation coefficients in serum and CSF samples in paired samples, *p* < 0.05 indicates significant difference [58]. ^c^*μ*mol/L. ^d^nmol/L. ^e^Wilcoxon signed rank test for paired samples. ^∗^*p* < 0.05; ^∗∗^*p* < 0.001.

**Table 3:** Associations between the frailty index and circulating metabolites in HUSK (*N* = 1691)^a^.

	Model 1: unadjusted^b^			Model 2: adjusted^c^		
Metabolites	*β*	[95% CI]	*p*	*β*	[95% CI]	*p*
CRP	0.19	[0.14, 0.22]	<.001^∗∗^	0.15	[0.11, 0.19]	<.001^∗∗^
Trp	<0.01	[−0.05, 0.04]	.765	0.01	[−0.04, 0.04]	.978
Kyn	0.15	[0.11, 0.19]	<.001^∗∗^	0.12	[0.07, 0.16]	<.001^∗∗^
HK	0.15	[0.10, 0.19]	<.001^∗∗^	0.11	[0.06, 0.15]	<.001^∗∗^
KA	0.08	[0.03, 0.12]	.001^∗^	0.07	[0.02, 0.11]	.003^∗^
AA	0.04	[−0.02, 0.06]	.068	0.03	[−0.01, 0.08]	.129
XA	0.02	[−0.02, 0.06]	.404	0.02	[−0.02, 0.07]	.322
HAA	0.10	[0.06, 0.15]	<.001^∗∗^	0.08	[0.04, 0.12]	<.001^∗∗^
PIC	−0.01	[−0.04, 0.04]	.950	0.01	[−0.03, 0.06]	.532
QA	0.18	[0.14, 0.22]	<.001^∗∗^	0.14	[0.10, 0.19]	<.001^∗∗^
KTR	0.15	[0.11, 0.19]	<.001^∗∗^	0.12	[0.07, 0.16]	<.001^∗∗^

Abbreviations: AA: anthranilic acid; CRP: C-reactive protein; HAA: 3-hydroxyanthranilic acid; HK: 3-hydroxykynurenine; KA: kynurenic acid; HUSK: Hordaland Health Study; Kyn: kynurenine; KTR: kynurenine to tryptophan ratio; *p*: *p* value; PIC: picolinic acid; Trp: tryptophan; XA: xanthurenic acid; QA: quinolinic acid; 95% CI: 95% confidence interval. ^a^The outcome, frailty, was calculated as a frailty index, multiplied by 100, log-transformed, and converted to a *z*-score. The standardized betas are reported, which should be interpreted similarly to correlation coefficients, as both the outcome and predictors were log-transformed. ^b^Linear regression model adjusted for age, sex, and smoking status. ^c^Linear regression model adjusted for age, sex, smoking status, and CRP (CRP adjusted for KTR). ^∗^*p* < 0.05; ^∗∗^*p* < .001.

## Data Availability

Some of the data in the study are available from the corresponding author upon request.

## Supplementary Materials

Supplementary Materials Supplementary materials includes a brief overview of the Melbourne Collaborative Cohort Study, The Hordaland Health Study, The COGNORM study, and the frailty index. Supplementary Figure 1. The Kynurenine: tryptophan ratio in plasma increases with age and over time. The vertical lines with confidence intervals represent increasing KTR with chronological age at baseline, whereas the upward-sloping lines represent the linear increase with time in the study in MCCS (community-dwelling persons) and WENBIT (patients with stable angina pectoris). Abbreviations: KTR: kynurenine to tryptophan ratio; MCCS: Melbourne Collaborative Cohort Study; WENBIT: Western Norway B Vitamin Intervention Trial. Supplementary Table 1. Components of the frailty index in the Hordaland Health Study. Abbreviations: BMI: body mass index; HADS, Hospital Anxiety and Depression Scale; m-MMSE: modified mini-mental state examination; GFR: glomerular filtration rate; WHR: waist-to-hip ratio. ^a^Upper arm circumference correlates moderately with lean mass as measured by dual-energy X-ray absorptiometry [58]. We assigned a simplified frailty score for sarcopenia based on the SD difference from the mean stratified by sex. ^b^A HADS score of ≥8 was an indicator of mild depressive symptoms, in accordance with Stern et al. [57]. ^c^The waist-hip ratio was defined in accordance with guidelines from the WHO [55]. ^d^Subjective cognitive symptom was not available in HUSK. Global *z*-scores from a neuropsychological test battery were scored from milder (>1SD) to more severe (<2SD) cognitive impairment to generate a frailty score with a simple scoring system. ^e^A modified, brief MMSE [56], where a score of ≤10 out of 12 indicated a possible abnormality as this was present in only 9.2% of the study population. Supplementary Table 2. Concentrations of metabolites and CRP in the four cohorts Note on concentrations: median and interquartile ranges are listed. The units of concentrations of metabolites are in nmol/L, except for tryptophan and kynurenine (*μ*mol/L). CRP is in *μ*g/ml. Abbreviations: AA: anthranilic acid; CSF: cerebrospinal fluid; HAA: 3-hydroxyanthranilic acid; HK: 3-hydroxykynurenine; KA: kynurenic acid; KTR: kynurenine to tryptophan ratio; Kyn: kynurenine; LOD: limit of detection; MCCS: Melbourne Collaborative Cohort Study; *p*: *p* value; PIC: picolinic acid; Trp: tryptophan; QA: quinolinic acid; WENBIT: Western Norway Vitamin B Intervention Trial; XA: xanthurenic acid. ^a^Measurements of Trp, kynurenines, and CRP in plasma. ^b^Thirty-three persons volunteered for repeat lumbar puncture after four years, with concentrations reported in results. Supplementary Table 3. Tryptophan, kynurenines, and aging: standardized effect size estimates. ^a^Note: standardized fixed effects of association between metabolites and age at baseline or change over time (years in study). The size of the effect can be interpreted similarly to correlation coefficients, with some limitations (see Statistics). Abbreviations: AA: anthranilic acid; HAA: 3-hydroxyanthranilic acid; HK: 3-hydroxykynurenine; KA: kynurenic acid; KTR: kynurenine to tryptophan ratio; Kyn: kynurenine; LLCI: lower level of 95% confidence interval; MCCS: Melbourne Collaborative Cohort Study; PIC: picolinic acid; sFE: standardized fixed effects; Trp: tryptophan; ULCI: upper level 95% confidence interval; QA: quinolinic acid; XA: xanthurenic acid; WENBIT: Western Norway B Vitamin Intervention Trial. ^a^Linear mixed-effects models. In MCCS, there were only two measurements, and thus a random intercept model was used. In WENBIT, there were three follow-ups, and a random coefficient model was used. Supplementary Table 4. Change in plasma metabolites according to a minor increment in age (one to three years) in participants from the HUSK cohort. ^a^Abbreviations: AA: anthranilic acid; CRP: C-reactive protein; GFR: glomerular filtration rate; HAA: 3-hydroxyanthranilic acid; HK: 3-hydroxykynurenine; KA: kynurenic acid; KTR: kynurenine to tryptophan ratio; Kyn: kynurenine; OR: odds ratio; *p*: *p* value; PIC: picolinic acid; Trp: tryptophan; QA: quinolinic acid; XA: xanthurenic acid; 95% CI: 95% confidence interval. ^a^The Hordaland Health Study (HUSK; *N* = 3161). Multinomial logistic regression with age 71 years as the reference, adjusted for sex. ^b^Ages included 71 (*n* = 566, reference group), 72 (*n* = 1088), 73 (*n* = 1055), and 74 (*n* = 452) years. ^c^Odds ratio from a multinomial model. ^∗^ < 0.05, ^∗∗^ < 0.001. Supplementary Table 5. Associations of kynurenines, and CRP with all-cause mortality. ^a^Note: Effect sizes indicate the hazard ratio associated with a one standard deviation increase in the log-transformed metabolite concentrations. Abbreviations: AA: anthranilic acid; 95% CI: 95% confidence interval; CRP: C-reactive protein; HAA: 3-hydroxyanthranilic acid; HK: 3-hydroxykynurenine; KA: kynurenic acid; KTR: kynurenine to tryptophan ratio; Kyn: kynurenine; PIC: picolinic acid; Trp: tryptophan; QA: quinolinic acid; XA: xanthurenic acid. ^a^Cox proportional hazard analysis with age, sex, glomerular filtration rate, body mass index, and smoking status (never, previous, current) as covariates in all models, with or without adjustment for CRP for the kynurenines, or KTR for CRP. ^b^HUSK: The Hordaland Health Studies (*N* = 3161); MCCS: Melbourne Collaborative Cohort Study (*N* = 970). ^c^CRP adjusted for KTR. Trp, Kyn, HK, KA, AA, XA, HAA, PIC, QA, and KTR adjusted for CRP. ^∗^*p* < 0.05; ^∗∗^*p* < .001.
