# Supplementary material for: Kynurenine Pathway Metabolites in the Blood and Cerebrospinal Fluid Are Associated with Human Aging
Source: Oxid Med Cell Longev. 2022 Oct 21;2022:5019752. doi: 10.1155/2022/5019752 (PMC9616658; doi:10.1155/2022/5019752)
Supplement: Supplementary Materials — includes a brief overview of the Melbourne Collaborative Cohort Study, The Hordaland Health Study, The COGNORM study, and the frailty index. Supplementary Figure 1. The Kynurenine: tryptophan ratio in plasma increases with age and over time. The vertical lines with confidence intervals represent increasing KTR with chronological age at baseline, whereas the upward-sloping lines represent the linear increase with time in the study in MCCS (community-dwelling persons) and WENBIT (patients with stable angina pectoris). Abbreviations: KTR: kynurenine to tryptophan ratio; MCCS: Melbourne Collaborative Cohort Study; WENBIT: Western Norway B Vitamin Intervention Trial. Supplementary Table 1. Components of the frailty index in the Hordaland Health Study. Abbreviations: BMI: body mass index; HADS, Hospital Anxiety and Depression Scale; m-MMSE: modified mini-mental state examination; GFR: glomerular filtration rate; WHR: waist-to-hip ratio. aUpper arm circumference correlates moderately with lean mass as measured by dual-energy X-ray absorptiometry [58]. We assigned a simplified frailty score for sarcopenia based on the SD difference from the mean stratified by sex. bA HADS score of ≥8 was an indicator of mild depressive symptoms, in accordance with Stern et al. [57]. cThe waist-hip ratio was defined in accordance with guidelines from the WHO [55]. dSubjective cognitive symptom was not available in HUSK. Global z-scores from a neuropsychological test battery were scored from milder (>1SD) to more severe (<2SD) cognitive impairment to generate a frailty score with a simple scoring system. eA modified, brief MMSE [56], where a score of ≤10 out of 12 indicated a possible abnormality as this was present in only 9.2% of the study population. Supplementary Table 2. Concentrations of metabolites and CRP in the four cohorts Note on concentrations: median and interquartile ranges are listed. The units of concentrations of metabolites are in nmol/L, except for t [file 5019752.f1.zip › Supplementary_materials (1).docx]

**SUPPLEMENTARY MATERIALS**

**Melbourne Collaborative Cohort Study**

The study participant were identified through electoral enrollment (compulsory) and phone directories. A strategy to enroll migrants included advertisements and community announcements ^1^. Participants were residents of the Melbourne metropolitan area (24,469 women and 17,044 men), with an average age of 55 years (range 27–76 years; 99% aged between 40 and 69). All were of White European origin; most (69%) were born in Australia or New Zealand, 13% were born in Italy, 11% in Greece, and 6% in the United Kingdom. Migrants were oversampled to increase genetic variation and extend the range of lifestyle exposures.

Vital status was ascertained through linkage of the cohort with the Victorian Registry of Births Deaths and Marriages through the Victorian Cancer Registry and the National Death Index through the Australian Institute of Health and Welfare, and considered to be virtually complete up to 31 October 2019.

**The Hordaland Health Study**

Participants were invited by letter, underwent a brief physical examination, completed self-administered questionnaires, and donated non-fasting blood samples. The self-administered questionnaire included questions relating to cardiovascular disease, diabetes mellitus, smoking habits, use of medications, physical exercise, and alcohol consumption. Blood pressure was recorded during the examination ^2^.

**The COGNORM study**

The COGNORM-study recruited 144 participants scheduled for elective gynecological, orthopedic, or urological surgery, aiming to assess CSF and magnetic resonance imaging in cognitively healthy older persons. Participants 65 years or older were recruited during 2012 and 2013 at Oslo University Hospital and Diakonhjemmet Hospital, Oslo, as described previously ^3^. Serum and CSF samples were transported on dry ice, centrifuged, alliquoted and stored at -80° C as the patients were included, with analyses conducted in 2020. Exclusion criteria were dementia, previous stroke with sequela, and known neurodegenerative disease. The CSF was sampled per-operatively prior to administration of spinal anesthesia ^3^. Participants were tested using a neuropsychological test battery.

However, not all participants were cognitively healthy at inclusion, and the present study

includes only cognitively healthy persons with paired CSF and serum samples (n = 109). Being cognitively healthy was defined as scoring >27 points on the Mini-Mental State Examination (MMSE) or scoring <27 on the MMSE with just one other abnormal test score (> 1.5 SD outside the age, education, and sex-based normal value) ^3^. After four years, all participants were invited for a second lumbar puncture, and 33 participants who consented and had available samples were included in this study.

##

## Frailty index

In a sub study of 2152 HUSK participants who underwent cognitive testing ^4^, we constructed a frailty index based on 38 various health conditions/candidate deficits. These included indicators of several chronic diseases, sarcopenia, mental health, social variables, and health behaviors, following the stepwise procedure described by Rockwood *et al*. ^5^, using cut-off values described in Supplementary Table 3 ^6-9^. The number of deficits recorded for each patient was divided by the total number of possible deficits (Supplementary Table 3), resulting in a potential range of 0 to 1. However, there were missing data for several participants. We excluded participants with missing information on six or more conditions. Thus, the frailty analyses included measurements from 1691 participants in HUSK and more than 32 health deficits for each participant.

**SUPPLEMENTARY BIBLIOGRAPHY**

**1.** Milne RL, Fletcher AS, MacInnis RJ, et al. Cohort Profile: The Melbourne Collaborative Cohort Study (Health 2020). *Int J Epidemiol.* 2017;46(6):1757-1757i. doi: 10.1093/ije/dyx085.

**2.** Refsum H, Nurk E, Smith AD, et al. The Hordaland Homocysteine Study: a community-based study of homocysteine, its determinants, and associations with disease. *J Nutr.* 2006;136(6 Suppl):1731s-1740s. doi: 10.1093/jn/136.6.1731S.

**3.** Idland AV, Sala-Llonch R, Borza T, et al. CSF neurofilament light levels predict hippocampal atrophy in cognitively healthy older adults. *Neurobiol Aging.* 2017;49:138-144. doi: 10.1016/j.neurobiolaging.2016.09.012.

**4.** Nurk E, Refsum H, Bjelland I, et al. Plasma free choline, betaine and cognitive performance: the Hordaland Health Study. *Br J Nutr.* 2013;109(3):511-519. doi: 10.1017/s0007114512001249.

**5.** Searle SD, Mitnitski A, Gahbauer EA, Gill TM, Rockwood K. A standard procedure for creating a frailty index. *BMC Geriatr.* 2008;8:24. doi: 10.1186/1471-2318-8-24.

**6.** Nishida C, Ko GT, Kumanyika S. Body fat distribution and noncommunicable diseases in populations: overview of the 2008 WHO Expert Consultation on Waist Circumference and Waist-Hip Ratio. *Eur J Clin Nutr.* 2010;64(1):2-5. doi: 10.1038/ejcn.2009.139.

**7.** Braekhus A, Laake K, Engedal K. The Mini-Mental State Examination: identifying the most efficient variables for detecting cognitive impairment in the elderly. *J Am Geriatr Soc.* 1992;40(11):1139-1145. doi: 10.1111/j.1532-5415.1992.tb01804.x.

**8.** Stern AF. The hospital anxiety and depression scale. *Occup Med (Lond).* 2014;64(5):393-394. doi: 10.1093/occmed/kqu024.

**9.** Diano D, Ponti F, Guerri S, et al. Upper and lower limbs composition: a comparison between anthropometry and dual-energy X-ray absorptiometry in healthy people. *Arch Osteoporos.* 2017;12(1):78. doi: 10.1007/s11657-017-0374-8.

**Supplementary Figure 1. The Kynurenine:tryptophan ratio in plasma increases with age and over time.** The vertical lines with confidence intervals represent increasing KTR with chronological age at baseline, whereas the upward-sloping lines represent the linear increase with time in the study in MCCS (Community- dwelling persons) and WENBIT (Patients with stable angina pectoris). Abbreviations: KTR, kynurenine to tryptophan ratio, MCCS, Melbourne Collaborative Cohort Study; WENBIT, Western Norway B Vitamin Intervention Trial.

| **Supplementary Table 1.** Components of the Frailty Index in the Hordaland Health Study | | |
| --- | --- | --- |
| Variables in Frailty Index |  | Cut point |
| Stroke |  | Yes = 1, No = 0 |
| Myocardial infarction |  | Yes = 1, No = 0 |
| Angina pectoris |  | Yes = 1, No = 0 |
| Hypertension |  | 180/110 mmHg = 1, 160/100 mmHg = 0,66, 140/90 mmHg = 0.33, No = 0 |
| Diabetes |  | Yes = 1, No = 0 |
| Hyperglycemia |  | Glucose >=11.1 = 1, Glucose >= 7.8 = 0.5, No = 0 |
| Renal failure |  | GFR <30 = 1, GFR <45 = 0.66, GFR <60 = 0.33, No = 0 |
| Asthma |  | Yes = 1, No = 0 |
| Bronchitis |  | Yes = 1, No = 0 |
| Sarcopenia^a^ |  | >2SD from the sex specific upper arm circumference mean =1,  >=1 – 2SD from the sex specific upper arm circumference mean = 0.5. |
| Depression ^b^ |  | HADS >= 8 p = 1, HADS <= 7 p = 0 |
| Osteoporosis |  | Yes = 1, No = 0 |
| Thromboembolism |  | Yes = 1, No = 0 |
| Previous cancer |  | Yes = 1, No = 0 |
| Upper limb fracture |  | Yes = 1, No = 0 |
| Lower limb fracture |  | Yes = 1, No = 0 |
| Body mass index |  | BMI <21 or >=33 = 1, BMI >21 or <33 = 0 |
| Waist-to-hip-ratio ^c^ |  | WHR >=0.9 (men) or WHR >=0.85 (women) = 1, otherwise 0 |
| Global cognition score ^d^ |  | Z-score <-2 = 1, Z-score <-1.5 = 0.75, Z-score <-1 = 0.5 |
| m-MMSE ^e^ |  | MMSE score <=10 =1, MMSE score >10 = 0 |
| Self-reported health overall |  | Very severe complaints = 1, Severe complaints = 0.66, Moderate complaints = 0.33, Low/none complaints = 0. |
| Self-reported health at present |  | Severe complaints = 1, Moderate complaints = 0.5 |
| Paresis |  | Three paresis (facial paresis, arm paresis and leg paresis) = 1, two paresis = 0.75, one paresis = 0.5, no paresis = 0 |
| Aphasia |  | Yes = 1, No = 0 |
| Dyspnea |  | Yes = 1, No = 0 |
| Morning cough |  | Yes = 1, No = 0 |
| Chest pain |  | Yes = 1, No = 0 |
| Leg pain when walking |  | Yes = 1, No = 0 |
| Pain when working |  | Yes = 1, No = 0 |
| Feeling lonely |  | Yes = 1, No = 0 |
| Social impairment |  | To a great extent = 1, To some extent = 0.5, Not at all = 0 |
| Any chest pain |  | Yes = 1, No = 0 |
| Exertional chest pain |  | Flat surfaces = 1, Stairs or slopes = 0.5, No = 0 |
| Exertional dyspnea |  | Yes = 1, No = 0 |
| Lack of physical exercise |  | Yes = 1, No = 0 |
| Dysphagia |  | Yes = 1, No = 0 |
| Dry mouth |  | Yes = 1, No = 0 |
| Dry eyes |  | Yes = 1, No = 0 |
| Abbreviations: BMI, body mass index; HADS, Hospital Anxiety and Depression Scale; m-MMSE, Modified Mini-Mental State Examination; GFR, glomerular filtration rate; WHR, waist-to-hip ratio.  ^a^ Upper arm circumference correlates moderately with lean mass as measured by dual-energy X-ray absorptiometry ^58^. We assigned a simplified frailty score for sarcopenia based on the SD difference from the mean stratified by sex.  ^b^ A HADS score of ≥ 8 was an indicator of mild depressive symptoms, in accordance with Stern et al. ^57^. ^c^ The waist-hip ratio was defined in accordance with guidelines from the WHO ^55^.  ^d^ Subjective cognitive symptom was not available in HUSK. Global z-scores from a neuropsychological test battery were scored from milder (>1SD) to more severe (<2SD) cognitive impairment to generate a frailty score with a simple scoring system.  ^e^ A modified, brief MMSE ^56^, where a score of <=10 out of 12 indicated a possible abnormality as this was present in only 9.2% of the study population. | | |

| **Supplementary Table 2.** Concentrations of metabolites and CRP in the four cohorts | | | | |
| --- | --- | --- | --- | --- |
| **MCCS: Community-dwelling persons (N = 970, Follow-up after 11 years)^a^** | | | | |
| Metabolites | **Baseline** | **Follow-up 1** | | **Follow-up 2** |
| CRP | 0.99 (1.88) | 1.07 (1.83) | |  |
| Trp | 65.9 (14.3) | 59.1 (15.6) | |  |
| Kyn | 1.53 (0.47) | 1.63 (0.53) | |  |
| HK | 38.7 (15.5) | 39.4 (17.5) | |  |
| KA | 48.1 (22.6) | 53.2 (26.5) | |  |
| AA | 20.4 (10.4) | 16.7 (6.7) | |  |
| XA | 15.0 (9.0) | 15.3 (9.6) | |  |
| HAA | 30.1 (15.3) | 32.2 (15.6) | |  |
| PIC | 30.3 (15.7) | 33.9 (17.9) | |  |
| QA | 378 (153) | 447 (230) | |  |
| KTR | 2.30 (0.72) | 2.75 (0.88) | |  |
| **WENBIT: Patients with stable angina pectoris (N = 604, follow-up after one and three years)**^a^ | | | | |
| CRP | 1.76 (2.82) | 1.62 (2.26) | | 1.46 (2.35) |
| Trp | 69.8 (18.4) | 67.1 (15.9) | | 66.9 (15.9) |
| Kyn | 1.66 (0.56) | 1.70 (0.58) | | 1.74 (0.54) |
| HK | 28.7 (13.7) | 31.3 (13.8) | | 31.1 (15.7) |
| KA | 48.9 (24.7) | 52.5 (27.1) | | 53.2 (27.7) |
| AA | 13.6 (7.2) | 14.7 (7.5) | | 14.6 (7.0) |
| XA | 14.1 (8.1) | 16.1 (10.2) | | 16.0 (9.6) |
| HAA | 34.6 (20.1) | 37.4 (20.7) | | 38.1 (20.5) |
| QA | 392 (166) | 418 (163) | | - |
| KTR | 2.37 (0.92) | 2.45 (0.88) | | 2.57 (0.92) |
| **Hordaland Health Study: Community-dwelling persons (N = 3161)^a^** | | | | |
| CRP | 4.0 (3.4) | | | |
| Trp | 69.1 (17.2) | | | |
| Kyn | 1.8 (0.49) | | | |
| HK | 38.9 (15.5) | | | |
| KA | 57.9 (25.5) | | | |
| AA | 17.5 (7.2) | | | |
| XA | 17.9 (9.8) | | | |
| HAA | 36.8 (17.5) | | | |
| PIC | 57.0 (28.8) | | | |
| QA | 510.3 (225) | | | |
| KTR | 2.60 (0.70) | | | |
| **COGNORM-study: Cognitively normal persons (N = 109)^b^** | | | | |
|  | **Serum** |  | **CSF** |  |
| Trp | 57.5 (15.2) |  | 2.81 (0.79) |  |
| Kyn | 1.58 (0.60) |  | 0.05(0.03) |  |
| HK | 48.9 (26.3) |  | 4.78 (3.62) |  |
| KA | 55.8 (34.0) |  | 3.04 (2.85) |  |
| AA | 17.6 (8.4) |  | 10.9 (6.8) |  |
| XA | 13.1 (7.6) |  | < LOD |  |
| HAA | 28.3 (13.5) |  | < LOD |  |
| PIC | 33.9 (14.8) |  | 20.9 (8.90) |  |
| QA | 419 (246) |  | 37.2 (21.0) |  |
| Note on concentrations: Median and (interquartile ranges) are listed. The units of concentrations of metabolites are in nmol/L, except for tryptophan and kynurenine (µmol/L). CRP is in µg/ml.  Abbreviations: AA, anthranilic acid; CSF, cerebrospinal fluid; HAA, 3-hydroxyanthranilic acid; HK, 3-hydroxykynurenine; KA, kynurenic acid; KTR, kynurenine to tryptophan ratio; Kyn, kynurenine; LOD; limit of detection; MCCS, Melbourne Collaborative Cohort Study; p, p-value; PIC, picolinic acid; Trp, tryptophan; QA, quinolinic acid; WENBIT, Western Norway Vitamin B Intervention Trial; XA, xanthurenic acid.  ^a^ Measurements of Trp, kynurenines, and CRP in plasma.  ^b^ Thirty-three persons volunteered for repeat lumbar puncture after four years, with concentrations reported in results. | | | | |

| **Supplementary Table 3.** Tryptophan, kynurenines, and aging: standardized effect size estimates^a^. | | | | | | | |
| --- | --- | --- | --- | --- | --- | --- | --- |
|  |  |  |  |  |  |  |  |
|  | **Age** | | |  | **Change over time** | | |
|  |  | | |  |  | | |
| **Community-dwelling persons (MCCS)** | | | | | | | |
|  | sFE | LLCI | ULCI |  | sFE | LLCI | ULCI |
| CRP | 0.15 | 0.10 | 0.20 |  | 0.02 | -0.02 | 0.05 |
| Trp | -0.10 | -0.15 | -0.06 |  | -0.31 | -0.35 | -0.27 |
| Kyn | 0.25 | 0.20 | 0.30 |  | 0.15 | 0.12 | 0.18 |
| HK | 0.20 | 0.15 | 0.26 |  | 0.06 | 0.03 | 0.09 |
| KA | 0.13 | 0.08 | 0.18 |  | 0.18 | 0.15 | 0.21 |
| AA | 0.13 | 0.08 | 0.18 |  | -0.28 | -0.31 | -0.24 |
| XA | -0.05 | -0.11 | 0.01 |  | -0.01 | -0.04 | 0.03 |
| HAA | 0.01 | -0.04 | 0.05 |  | 0.16 | 0.12 | 0.20 |
| QA | 0.32 | 0.27 | 0.37 |  | 0.26 | 0.23 | 0.28 |
| KTR | 0.29 | 0.24 | 0.34 |  | 0.35 | 0.32 | 0.38 |
|  |  |  |  |  |  |  |  |
| **Patients with stable angina pectoris (WENBIT)** | | | | | | | |
| CRP | 0.01 | -0.06 | 0.07 |  | -0.04 | -0.07 | -0.01 |
| Trp | -0.15 | -0.22 | -0.09 |  | -0.06 | -0.10 | -0.02 |
| Kyn | 0.33 | 0.26 | 0.39 |  | 0.09 | 0.06 | 0.12 |
| HK | 0.22 | 0.17 | 0.28 |  | 0.07 | 0.04 | 0.10 |
| KA | 0.20 | 0.14 | 0.27 |  | 0.12 | 0.09 | 0.14 |
| AA | 0.26 | 0.19 | 0.33 |  | 0.06 | 0.03 | 0.09 |
| XA | 0.01 | -0.05 | 0.08 |  | 0.10 | 0.07 | 0.14 |
| HAA | -0.02 | -0.09 | 0.05 |  | 0.10 | 0.07 | 0.13 |
| QA | 0.36 | 0.30 | 0.43 |  | 0.20 | 0.13 | 0.27 |
| KTR | 0.39 | 0.32 | 0.45 |  | 0.12 | 0.09 | 0.14 |
| Note: Standardized fixed effects of association between metabolites and age at baseline or change over time (years in study). The size of the effect can be interpreted similarly to correlation coefficients, with some limitations (see Statistics).  Abbreviations: AA, anthranilic acid; HAA, 3-hydroxyanthranilic acid; HK, 3-hydroxykynurenine; KA, kynurenic acid; KTR, kynurenine to tryptophan ratio; Kyn, kynurenine; LLCI, lower level of 95% confidence interval; MCCS, Melbourne Collaborative Cohort Study; PIC, picolinic acid; sFE, standardized fixed effects; Trp, tryptophan; ULCI, upper level 95% confidence interval; QA, quinolinic acid; XA, xanthurenic acid; WENBIT, Western Norway B Vitamin Intervention Trial.  ^a^ Linear mixed-effects models. In MCCS, there were only two measurements, and thus a random intercept model was used. In WENBIT, there were three follow-ups, and a random coefficient model was used. | | | | | | | |

| **Supplementary Table 4.** Change in plasma metabolites according to a minor increment in age (one to three years) in participants from the HUSK cohort a | | | | |
| --- | --- | --- | --- | --- |
| Metabolites | Age (years)b | ORc | 95% CI | p |
| CRP | 72 | 1.08 | [0.97, 1.20] | .142 |
|  | 73 | 1.07 | [0.96, 1.19] | .241 |
|  | 74 | 1.12 | [0.98, 1.27] | .084 |
| Trp | 72 | 0.91 | [0.82, 1.00] | .055 |
|  | 73 | 0.93 | [0.84, 1.03] | .145 |
|  | 74 | 0.86 | [0.76, 0.98] | .019* |
| Kyn | 72 | 1.01 | [0.92, 1.12] | .735 |
|  | 73 | 1.00 | [0.90, 1.11] | .988 |
|  | 74 | 1.03 | [0.91, 1.16] | .681 |
| HK | 72 | 1.09 | [0.98, 1.21] | .114 |
|  | 73 | 1.14 | [1.02, 1.26] | .016* |
|  | 74 | 1.26 | [1.11, 1.42] | <.001** |
| KA | 72 | 1.04 | [0.94, 1.15] | .476 |
|  | 73 | 1.04 | [0.94, 1.15] | .479 |
|  | 74 | 1.12 | [0.99, 1.27] | .071 |
| AA | 72 | 0.98 | [0.88, 1.08] | .653 |
|  | 73 | 1.01 | [0.91, 1.12] | .876 |
|  | 74 | 1.01 | [0.90, 1.15] | .842 |
| XA | 72 | 0.94 | [0.85, 1.04] | .252 |
|  | 73 | 0.95 | [0.86, 1.05] | .311 |
|  | 74 | 0.92 | [0.81, 1.04] | .160 |
| HAA | 72 | 0.99 | [0.89, 1.10] | .883 |
|  | 73 | 1.10 | [0.99, 1.22] | .072 |
|  | 74 | 1.08 | [0.95, 1.22] | .227 |
| PIC | 72 | 0.97 | [0.88, 1.08] | .585 |
|  | 73 | 1.00 | [0.90, 1.10] | .944 |
|  | 74 | 1.00 | [0.88, 1.13] | .981 |
| QA | 72 | 1.13 | [1.02, 1.25] | .022* |
|  | 73 | 1.12 | [1.01, 1.24] | .038* |
|  | 74 | 1.16 | [1.02, 1.31] | .020* |
| KTR | 72 | 1.10 | [0.99, 1.22] | .072 |
|  | 73 | 1.07 | [0.97, 1.19] | .194 |
|  | 74 | 1.17 | [1.03, 1.33] | .012* |
| Abbreviations: AA, anthranilic acid; CRP, C-reactive protein; GFR, glomerular filtration rate; HAA, 3-hydroxyanthranilic acid; HK, 3-hydroxykynurenine; KA, kynurenic acid; KTR, kynurenine to tryptophan ratio; Kyn, kynurenine; OR, odds ratio; p, p- value; PIC, picolinic acid; Trp, tryptophan; QA, quinolinic acid; XA, xanthurenic acid; 95% C.I, 95% confidence interval.  ^a^ The Hordaland Health Study (HUSK; N = 3161). Multinomial logistic regression with age 71 years as the reference, adjusted for sex.  ^b^ Ages included 71 (n = 566, reference group), 72 (n = 1088), 73 (n = 1055) and 74 (n = 452) years.  ^c^ Odds ratio from a multinomial model.  * < 0.05, ** < 0.001. | | | | |

| **Supplementary Table 5.** Associations of kynurenines, and CRP with all-cause mortalitya | | | | | | | | |
| --- | --- | --- | --- | --- | --- | --- | --- | --- |
| Metabolites | Cohort^b^ | HR | 95% CI | p |  | HR | 95% CI | p |
|  |  |  | Unadjusted for KTR |  |  |  | Adjusted for KTR^c^ | |
| CRP | HUSK | 1.14 | [1.09, 1.20] | <.001** |  | 1.09 | [1.03, 1.15] | .001* |
|  | MCCS | 1.21 | [1.09, 1.34] | .001* |  | 1.20 | [1.08, 1.34] | .001* |
|  |  |  | Unadjusted for CRP |  |  |  | Adjusted for CRP^c^ | |
| Trp | HUSK | 0.89 | [0.84, 0.94] | <.001** |  | 0.90 | [0.85, 0.95] | <.001** |
|  | MCCS | 0.87 | [0.79, 0.97] | .012* |  | 0.87 | [0.79, 0.97] | .010* |
| Kyn | HUSK | 1.14 | [1.08, 1.22] | <.001** |  | 1.11 | [1.05, 1.18] | .001* |
|  | MCCS | 0.99 | [0.86, 1.27] | .835 |  | 0.96 | [0.84, 1.10] | .561 |
| HK | HUSK | 1.16 | [1.10, 1.22] | <.001** |  | 1.13 | [1.07, 1.20] | <.001** |
|  | MCCS | 1.09 | [0.96, 1.25] | .178 |  | 1.07 | [0.93, 1.22] | .336 |
| KA | HUSK | 1.06 | [0.99, 1.12] | .077 |  | 1.05 | [0.99, 1.12] | .121 |
|  | MCCS | 1.06 | [0.95, 1.20] | .301 |  | 1.07 | [0.95, 1.20] | .252 |
| AA | HUSK | 1.12 | [1.06, 1.17] | <.001** |  | 1.14 | [1.08, 1.19] | <.001** |
|  | MCCS | 1.13 | [1.01, 1.27] | .047* |  | 1.11 | [0.98, 1.26] | .079 |
| XA | HUSK | 0.92 | [0.87, 0.97] | .001* |  | 0.93 | [0.88, 0.98] | .008* |
|  | MCCS | 0.96 | [0.86, 1.08] | .507 |  | 0.97 | [0.86, 1.09] | .605 |
| HAA | HUSK | 0.97 | [0.92, 1.02] | .232 |  | 0.96 | [0.91, 1.01] | .142 |
|  | MCCS | 1.10 | [0.97, 1.25] | .140 |  | 1.07 | [0.94, 1.21] | .337 |
| PIC | HUSK | 1.02 | [0.97, 1.08] | .374 |  | 1.03 | [0.98, 1.08] | .295 |
|  | MCCS | 1.08 | [0.96, 1.21] | .203 |  | 1.06 | [0.94, 1.18] | .336 |
| QA | HUSK | 1.19 | [1.13, 1.26] | <.001** |  | 1.17 | [1.10, 1.24] | <.001** |
|  | MCCS | 1.32 | [1.15, 1.51] | <.001** |  | 1.28 | [1.12, 1.47] | <.001** |
| KTR | HUSK | 1.27 | [1.21, 1.34] | <.001** |  | 1.25 | [1.18, 1.32] | <.001** |
|  | MCCS | 1.14 | [1.01, 1.29] | .035* |  | 1.13 | [1.01, 1.28] | .047* |
| Note: Effect sizes indicate the hazard ratio associated with a one standard deviation increase in the log-transformed metabolite concentrations.  Abbreviations: AA, anthranilic acid; CI, 95% confidence interval; CRP, C-reactive protein; HAA, 3-hydroxyanthranilic acid; HK, 3-hydroxykynurenine; KA, kynurenic acid; KTR, kynurenine to tryptophan ratio; Kyn, kynurenine; PIC, picolinic acid; Trp, tryptophan; QA, quinolinic acid; XA, xanthurenic acid; 95%  ^a^ Cox proportional hazard analysis with age, sex, glomerular filtration rate, body-mass index and smoking status (never, previous, current) as covariates in all models, with or without adjustment for CRP for the kynurenines, or KTR for CRP.  ^b^ HUSK, The Hordaland Health Studies (N = 3161); MCCS, Melbourne Collaborative Cohort Study (N = 970).  ^c^ CRP adjusted for KTR. Trp, Kyn, HK, KA, AA, XA, HAA, PIC, QA, and KTR adjusted for CRP.  * p < 0.05; ** p < .001. | | | | | | | | |
