# Supplementary figures and images for: Kynurenine Pathway Metabolites in the Blood and Cerebrospinal Fluid Are Associated with Human Aging
Source: Oxid Med Cell Longev. 2022 Oct 21;2022:5019752. doi: 10.1155/2022/5019752 (PMC9616658; doi:10.1155/2022/5019752)

A: Community-dwelling persons (N = 970)

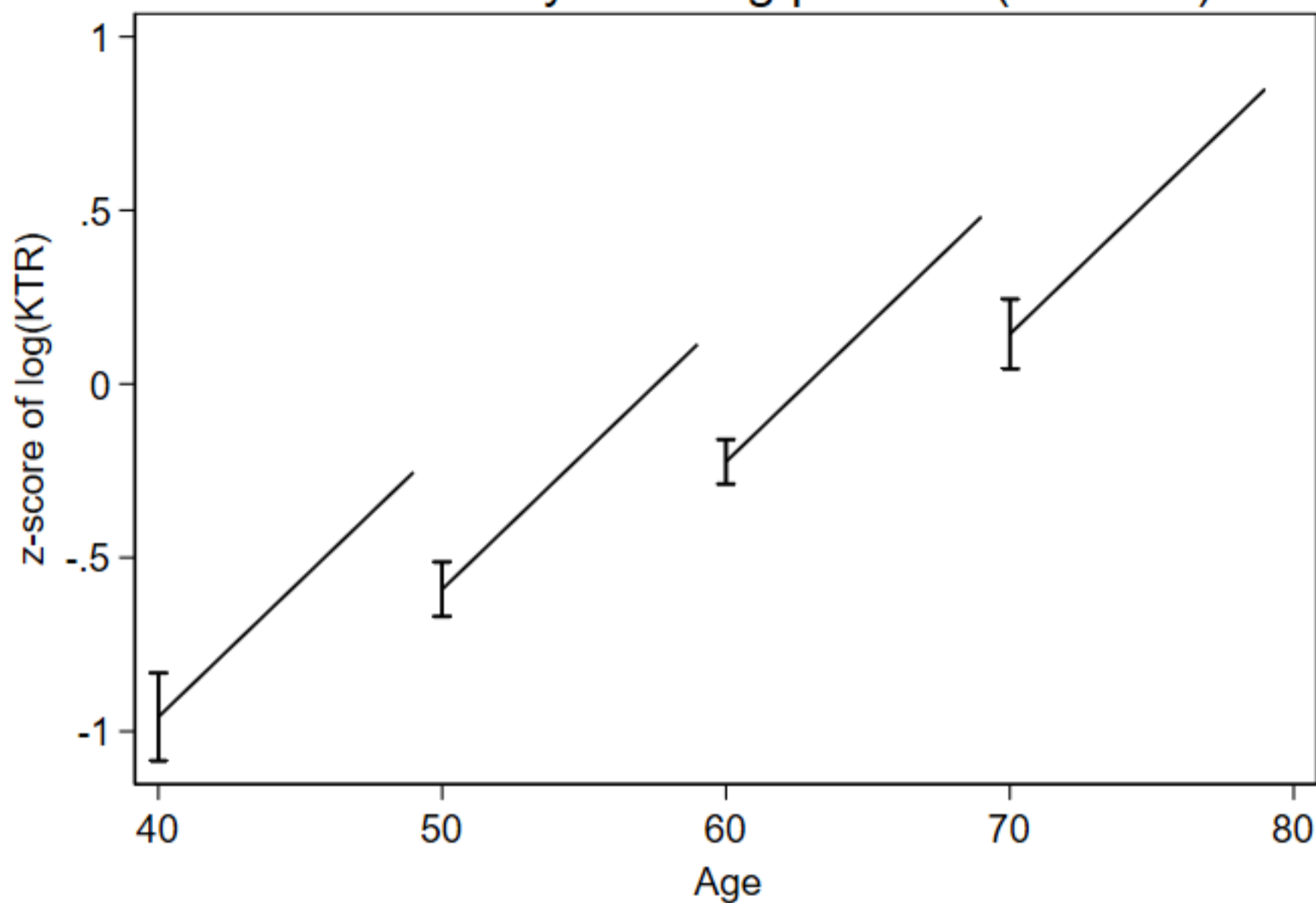

B: Patients with stable angina pectoris (N = 604)

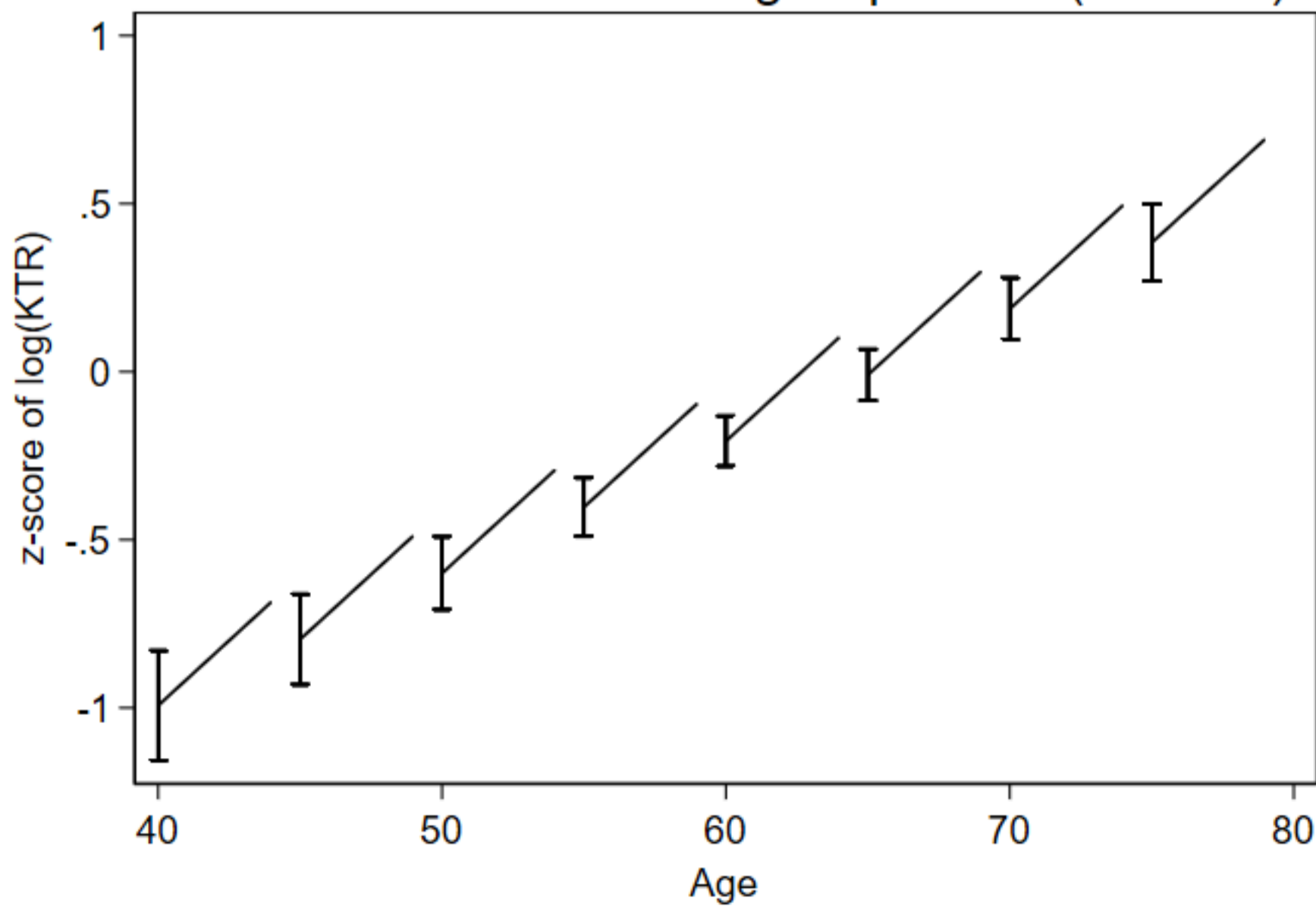

Supplement: Supplementary Materials — includes a brief overview of the Melbourne Collaborative Cohort Study, The Hordaland Health Study, The COGNORM study, and the frailty index. Supplementary Figure 1. The Kynurenine: tryptophan ratio in plasma increases with age and over time. The vertical lines with confidence intervals represent increasing KTR with chronological age at baseline, whereas the upward-sloping lines represent the linear increase with time in the study in MCCS (community-dwelling persons) and WENBIT (patients with stable angina pectoris). Abbreviations: KTR: kynurenine to tryptophan ratio; MCCS: Melbourne Collaborative Cohort Study; WENBIT: Western Norway B Vitamin Intervention Trial. Supplementary Table 1. Components of the frailty index in the Hordaland Health Study. Abbreviations: BMI: body mass index; HADS, Hospital Anxiety and Depression Scale; m-MMSE: modified mini-mental state examination; GFR: glomerular filtration rate; WHR: waist-to-hip ratio. aUpper arm circumference correlates moderately with lean mass as measured by dual-energy X-ray absorptiometry [58]. We assigned a simplified frailty score for sarcopenia based on the SD difference from the mean stratified by sex. bA HADS score of ≥8 was an indicator of mild depressive symptoms, in accordance with Stern et al. [57]. cThe waist-hip ratio was defined in accordance with guidelines from the WHO [55]. dSubjective cognitive symptom was not available in HUSK. Global z-scores from a neuropsychological test battery were scored from milder (>1SD) to more severe (<2SD) cognitive impairment to generate a frailty score with a simple scoring system. eA modified, brief MMSE [56], where a score of ≤10 out of 12 indicated a possible abnormality as this was present in only 9.2% of the study population. Supplementary Table 2. Concentrations of metabolites and CRP in the four cohorts Note on concentrations: median and interquartile ranges are listed. The units of concentrations of metabolites are in nmol/L, except for t [file 5019752.f1.zip › Supplementary_Figure1_age_ktr.pdf]
